# A Snakebite Cured

**Published:** 1889-06-22

**Authors:** 


### A SNAKEBITE CURED.

Mr. Whitty, surgeon on board the "Roslin Castle," relates the following story in the *British Medical Journal*: On April 25, about 9.45 p.m., while on a voyage from the Cape, a gentleman brought a large puff adder into the smoking-room to exhibit to some of the other passengers. The snake was more than a yard long and as thick as a man's wrist. Suddenly the animal wriggled out of its box, and whilst its owner was trying to put it back again it bit him on the back of the right forefinger. Although bitten, he still stuck to the creature and struggled with it for ten minutes before he got it safely returned to its box. Ten minutes of invaluable time were thus lost before treatment was commenced. Dr. Whitty, so soon as the snake was secured from doing further harm, at once placed a tourniquet in position above the elbow to check if possible the flow of the virus in the direction of the upper arm and body by the bloodvessels or lymphatics. He then made a deep incision into the wound, and applied very freely compound camphor liniment, because he had no strong ammonia at hand. The hand, thus treated, was placed in hot water mixed with permangate of potash, in order that free bleeding might be encouraged and the poison thus expelled. Internally, two ounces of brandy and carbonate of ammonia with sulphuric æther were administered every ten minutes until four doses had been taken. That was the treatment. For the first ten hours the patient was in a most alarming and dangerous condition. Twenty minutes after the bite he fainted, but soon recovered. Then he became delirious and so restless that the temperature could not be taken. The hand and arm swelled, and were hard and brawny to the touch; the bitten finger turned quite black and as cold as ice. The pulse was at first quick, full, and bounding, but afterwards became slower and weaker. The patient continually made a "champing" noise with his mouth and tongue. By-and-by a kind of coma-vigil set in, the eyes were glazed, the pulse fell, and the patient was apparently very near death. Half an ounce of brandy was then given every half hour, with the result that the pulse was strengthened, and life gradually returned. The strength continued to alternate up and down for hours. Then vomiting set in, after which he was given another dose of brandy, and fell into a sound sleep. At half-past seven in the morning the dangerous symptoms were all past; and though the patient was much shaken for several days he left the ship at the end of the voyage comparatively well. So far as appears, Mr. Whitty's prompt local treatment and free use of stimulants undoubtedly saved his life.

